# Aprepitant for the management of nausea with inpatient IV dihydroergotamine

**DOI:** 10.1212/WNL.0000000000003206

**Published:** 2016-10-11

**Authors:** Denise E. Chou, Amy R. Tso, Peter J. Goadsby

**Affiliations:** From the Headache Center (D.E.C.), Department of Neurology, Columbia University Medical Center, New York, NY; Headache Group (D.E.C., P.J.G.), Department of Neurology, University of California, San Francisco; and Headache Group (A.R.T., P.J.G.), Basic & Clinical Neuroscience, and NIHR–Wellcome Trust King's Clinical Research Facility, King's College London, UK.

## Abstract

**Objective:**

To assess the efficacy and tolerability of oral aprepitant, a substance P/neurokinin A receptor antagonist, in controlling nausea associated with IV dihydroergotamine (DHE) administered for medically refractory migrainous headache in patients not responding to standard antiemetics or with a history of uncontrolled nausea with DHE.

**Methods:**

This was a retrospective chart review of prospectively collected hourly diary data and clinical notes of patients hospitalized between 2011 and 2015 for inpatient treatment with DHE. Patients were classified using the *International Classification of Headache Disorders*, 3rd edition (beta version). Peak and average daily nausea scores from hourly diaries, or daily entries of notes, and concurrent antiemetic use were collected and tabulated.

**Results:**

Seventy-four patients, of whom 24 had daily diaries, with chronic migraine with or without aura, with or without medication overuse, or new daily persistent headache of a migrainous type, were identified. In 36 of 57 cases in which aprepitant was administered during hospitalization, there was a 50% reduction in the average daily number of as-needed antinausea medications. Of 57 patients, 52 reported that the addition of aprepitant improved nausea. Among 21 of 24 patients with hourly diary data, nausea scores were reduced and in all 12 with vomiting there was cessation of emesis after aprepitant was added. Aprepitant was well tolerated with no treatment emergent adverse events.

**Conclusions:**

Aprepitant can be effective in the treatment of refractory DHE-induced nausea and emesis. Given the broader issue of troublesome nausea and vomiting in acute presentations of migraine, general neurologists may consider what place aprepitant has in the management of such patients.

**Classification of evidence:**

This study provides Class IV evidence that for patients with medically refractory migraine receiving IV DHE, oral aprepitant reduces nausea.

IV dihydroergotamine (DHE) has been established as an effective acute treatment for refractory migraine.^1,2^ The most common side effect of DHE is nausea, and its control is associated with better treatment outcomes.^2,3^ Indeed, nausea and vomiting control is a practical and common issue in emergency departments in the management of acute migraine.

Typically, premedication regimens used to prevent DHE-associated nausea, or indeed nausea more generally in migraine, target dopamine, serotonin 5-hydroxytryptamine (5-HT_3_), and histamine receptors. Aprepitant is a selective, high-affinity substance P/neurokinin-1 (NK_1_) receptor antagonist, which mitigates the emetic effects of substance P.^4^ Aprepitant has been used in the prevention of postoperative and chemotherapy-induced nausea and vomiting.^5^ In patients for whom conventional antiemetics were not effective, we began to use aprepitant and here report our experience with this novel approach to DHE-associated nausea.

## METHODS

We conducted a retrospective review of patients with migrainous disorders who were admitted to the University of California, San Francisco Headache Center for a 5-day course of IV DHE (cumulative dose of 11.25 mg) from June 1, 2011, through April 17, 2015, and received oral aprepitant as antiemetic therapy due to refractory nausea, either at the onset of DHE administration or later during their treatment course. The primary research question was whether aprepitant reduced nausea in patients receiving DHE for the treatment of medically refractory migraine. Aprepitant was given as a loading dose of 125 mg on day 1 and thereafter at 80 mg daily 30 minutes before the first daily dose of DHE.

### Standard protocol approvals, registrations, and patient consents.

The review was approved by the institutional review board of the University of California, San Francisco (12-09318).

### Data collection.

Peak and average daily nausea scores were determined before and after aprepitant administration from patients' hourly diaries, whereby headache and nausea were rated on an 11-point visual analog graph, or with daily progress notes, or both. Daily medication administration records were reviewed for other concurrent antiemetic medications given either as standing premedications or as-needed doses for breakthrough nausea.

### Efficacy assessment.

The efficacy of aprepitant was assessed by determining reduction (>50%) in either average or peak daily nausea score (1 point), cessation of emesis following administration of aprepitant, and ≥50% reduction in the average daily number of as-needed antinausea medications. In addition, patients were surveyed for their subjective impression regarding the effectiveness of aprepitant in controlling nausea, vomiting, or both with an open-ended question.

### Tolerability and adverse events.

Patient daily notes and diaries were reviewed for an assessment of any adverse events and to determine tolerability during inpatient use.

### Data collection and analysis.

Data were abstracted from patient hourly diaries or medical records into a summary spreadsheet (Excel; Microsoft, Redmond, WA). Summary data are presented as proportions observed. Given the exploratory nature of the work, the add-on nature of the treatment, and the absence of a suitable control, we did not have a formal aim for hypothesis testing.

## RESULTS

Seventy-four cases were identified (figure) with admission diagnoses, made by the authors and all checked by the senior author, of chronic migraine with or without aura, with or without medication overuse, or new daily persistent headache of a migrainous type.^6^

**Figure:**
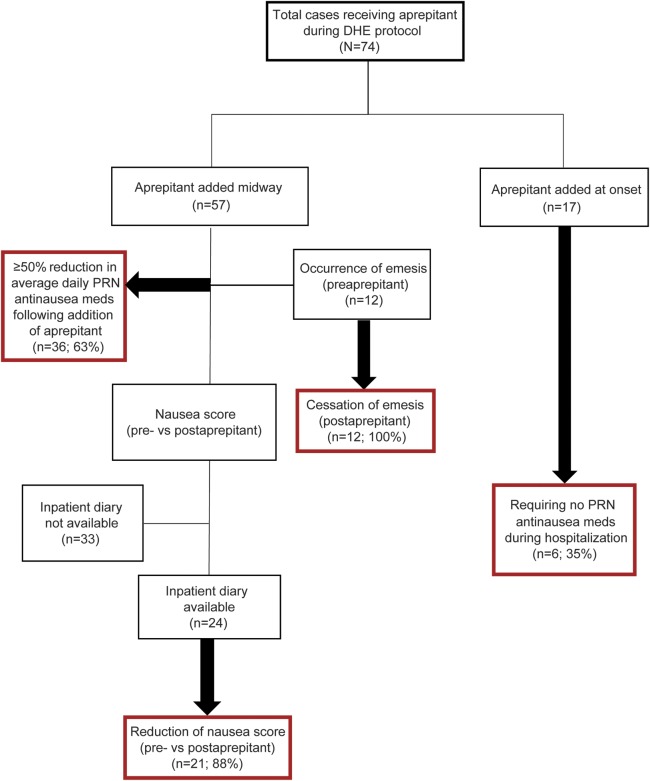
Flowchart of cases DHE = dihydroergotamine; PRN = as-needed.

### Timing of use and concomitant antiemetics.

In 57 of these cases, aprepitant was administered midway through the IV DHE protocol for nausea that was refractory to other standing premedication antiemetics, including IV ondansetron, granisetron, oral domperidone, or IV promethazine. In the remaining 17 cases, aprepitant was initiated on admission because of the presence of severe nausea at baseline or known history of severe nausea with prior DHE treatment. Inpatient diaries recording hourly nausea scores were available for review in 24 of the first 57 cases.

### Daily diary outcomes.

Mean or peak daily nausea scores, or both, were reduced following administration of aprepitant in 21 of the 24 patients (88%) whose diaries were available for review. Vomiting occurred in 12 patients before receiving aprepitant and ceased in all 12 cases post aprepitant. One patient developed emesis only after aprepitant was added.

### Aprepitant outcome when added during admission.

Among 36 of the 57 patients (63%) who received aprepitant midway through hospitalization, there was a ≥50% reduction in the average daily number of as-needed antinausea medications post aprepitant. Finally, 52 of the 57 patients (91%) reported subjective improvement of their nausea following the addition of aprepitant. Two reported no benefit and data were unavailable for the remaining 3 patients.

### Aprepitant at the onset of DHE.

Among the patients who received aprepitant at the onset of the DHE protocol (n = 17), 6 patients (35%) did not require any as-needed antinausea medications throughout the entire 5-day hospitalization. In these cases, other standing premedication nausea treatments given in addition to aprepitant on admission included ondansetron with domperidone (n = 4) or granisetron with domperidone (n = 1). Aprepitant was the sole premedication agent given in 1 of the 6 cases not requiring additional as-needed medications.

### Aprepitant and cannabis.

We noted a subgroup of patients withdrawn from daily cannabis use during their hospitalization for inpatient DHE (n = 7). Nausea was noted to be particularly severe in these cases, and refractory to domperidone and IV ondansetron. All patients demonstrated a response in at least 2 of the 3 outcome measures with addition of aprepitant.

### Tolerability and adverse events.

No adverse effects that could be attributable to aprepitant were reported by any of the 74 patients who received the medication. The medicine was extremely well-tolerated.

## DISCUSSION

These data demonstrate that aprepitant can provide relief of DHE-induced nausea and emesis where standard strategies, such as dopamine receptor antagonists, serotonin 5-HT_3_ receptor antagonists, and antihistamines, have failed. Moreover, aprepitant can be effective in patients with marked nausea before DHE administration and in patients with a history of DHE-induced nausea. A distinctive subgroup of patients with refractory nausea withdrawing from daily cannabinoid agonist use also respond well to aprepitant. While comparative efficacy studies would be appropriate to ascertain which is the best initial antiemetic path when using DHE, our data support the addition of aprepitant in patients whose conventional antiemetics failed or in those with special cases likely to be refractory to conventional antiemetics. Moreover, given the very common occurrence of nausea and vomiting in patients with migraine attending emergency departments and the excellent tolerability of aprepitant including a lack of sedative effects, its broader use could be considered in such settings.

DHE has been used in the treatment of migraine for many years.^7^ Nausea in association with administration of DHE is well recognized^1^; it has been suggested that its control is associated with better medium-term efficacy outcomes.^2,3^ Frequently used antiemetic medications in the context of DHE include ondansetron and granisetron (5-HT_3_ receptor antagonists), metoclopramide (dopamine D_2_ receptor antagonist), promethazine (primarily H_1_ and muscarinic acetylcholine receptor antagonist), prochlorperazine (primarily D_2_ receptor antagonist), and domperidone (peripheral D_2_ and D_3_ receptor antagonist). While domperidone and the 5-HT_3_ receptor antagonists are very well tolerated when used conservatively, metoclopramide, prochlorperazine, and promethazine are not without their own adverse events that can complicate the inpatient management of migraine. Despite vigorous treatment, some patients may develop significant nausea resulting in dose adjustments of DHE or premature termination of treatment with diminished efficacy.^2,8^ Aprepitant was developed for cancer therapy–induced emetogenesis^9^ and thus offers a novel approach to DHE-induced refractory nausea.

Substance P is a peptide of the neurokinin family, active at the NK_1_ receptor, which is found in vagal afferent nerves within the gastrointestinal tract and in regions of the CNS that are involved in control of the vomiting reflex: the nucleus tractus solitarius and area postrema.^4^ Aprepitant is a potent NK_1_ receptor antagonist.^5^ Human PET studies show that aprepitant crosses the blood–brain barrier and acts on NK_1_ receptors, with minimal affinity for NK_2_ and NK_3_, serotonin 5-HT_3_, dopamine, or corticosteroid receptors.^10^ In clinical use, aprepitant is very well tolerated with generally mild side effects reported in cancer studies.^9^ Two notable drug interactions are that barbiturates may reduce aprepitant blood levels and aprepitant may induce estrogen metabolism, thereby affecting contraception in the menstrual cycle in which it is used.

An important limitation of this report is the lack of blinding or a contemporaneous control group. Given the data we now have, a blinded study is feasible. The use of placebo is more complex since aprepitant is an established agent for nausea and emesis, albeit in cancer. Comparative efficacy studies are certainly possible, such as comparing ondansetron and aprepitant.

Herein, we provide data suggesting that aprepitant can be effective in DHE-induced nausea and emesis where usual treatment had failed, even in patients withdrawing from cannabinoids. Given the excellent tolerability of aprepitant, it seems reasonable to consider its use in refractory DHE-induced nausea and emesis, in particular, and more broadly in migraine-associated nausea and vomiting.

## GLOSSARY

DHE: dihydroergotamine
5-HT_3_: 5-hydroxytryptamine
NK_1_: neurokinin-1
